# Supplementary material for: Individual and population level costs and health-related quality of life outcomes of third-generation cephalosporin resistant bloodstream infection in Blantyre, Malawi
Source: PLOS Glob Public Health. 2023 Jun 22;3(6):e0001589. doi: 10.1371/journal.pgph.0001589 (PMC10287011; doi:10.1371/journal.pgph.0001589)
Supplement: S1 Text — (DOCX) [file pgph.0001589.s001.docx]

**S1 Text: Methods**

The microbiology surveillance data for Blantyre (available for 1998-2016 for adults and children)(20) were used in conjunction with historical and projected population data for Blantyre and Malawi (28), to estimate the annual number of cases for 3GC-R and 3GC-S *E. coli* and *Klebsiella* BSI. S1 Table, shows the total population for Blantyre and Malawi obtained from Malawi National Statistics Office(28) and S2 Table, shows the observed and estimated annual cases of 3GC-S and 3GC-R *E. coli* and *Klebsiella* BSI in Blantyre and Malawi.

In order to make projections, a number of assumptions were made. First, it was assumed that the incidence of BSI in Blantyre reflects the incidence in Malawi as a whole. Second, to make projections beyond 2016, it was assumed that the rate of increase in incidence of infections remained constant at 2016 levels. Third, the direct medical, societal costs and deterioration in HRQoL associated with hospitalisation for BSI in children was assumed to be equivalent to that estimated for the adults.

To estimate the total annual direct medical and societal costs, the estimated cost per case of BSI obtained from the economic outputs as described in the Methods, was multiplied by the number of estimated annual cases.

To estimate the annual QALYs lost, total QALYs lost amongst those who died during hospitalisation were added to total QALYs lost during the acute illness of survivors.

To estimate the QALYs lost amongst those who died, mortality rates observed in the hospital cohort were used. QALYs lost were modelled at the population level (including adults and children), and it was assumed that a death from BSI resulted in a loss of 45 QALYs, that BSI cases were equally distributed amongst children and adults, and that mortality rates between children and adults were comparable. To estimate the QALYs lost amongst those who survived, the EQ-5D utility scores described in the methods were used, and this detriment in HRQoL was assumed to last for approximately one month. The estimation of total annual QALYS lost used annual estimated cases for the *E. coli* and *Klebsiella* spp., as shown in S5
