# Supplementary material for: Individual and population level costs and health-related quality of life outcomes of third-generation cephalosporin resistant bloodstream infection in Blantyre, Malawi
Source: PLOS Glob Public Health. 2023 Jun 22;3(6):e0001589. doi: 10.1371/journal.pgph.0001589 (PMC10287011; doi:10.1371/journal.pgph.0001589)
Supplement: S1 Table — (DOCX) [file pgph.0001589.s002.docx]

S1 Table

S1 Table: Historical and projection population for Blantyre and Malawi.

| Year | Blantyre Population (all ages) | Malawi Population (all ages) |  |
| --- | --- | --- | --- |
|  |  |  |  |
| 1998 | 499,000 | 9,933,868 |  |
| 1999 | 514,000 | 10,152,753 |  |
| 2000 | 529,000 | 10,475,257 |  |
| 2001 | 544,000 | 10,816,294 |  |
| 2002 | 559,000 | 11,174,648 |  |
| 2003 | 575,000 | 11,548,841 |  |
| 2004 | 592,000 | 11,937,934 |  |
| 2005 | 609,000 | 12,341,170 |  |
| 2006 | 626,000 | 12,757,883 |  |
| 2007 | 644,000 | 13,187,632 |  |
| 2008 | 663,000 | 13,077,160 |  |
| 2009 | 682,000 | 13,512,376 |  |
| 2010 | 701,000 | 13,947,592 |  |
| 2011 | 721,000 | 14,388,550 |  |
| 2012 | 742,000 | 14,844,822 |  |
| 2013 | 763,000 | 15,316,860 |  |
| 2014 | 785,000 | 15,813,646 |  |
| 2015 | 808,000 | 16,310,431 |  |
| 2016 | 831,000 | 16,859,977 |  |
| 2017 | 855,000 | 17,409,522 |  |
| 2018 | 879,000 | 17,959,068 |  |
| 2019 | 905,000 | 18,508,613 |  |
| 2020 | 932,000 | 19,104,275 |  |
| 2021 | 962,000 | 19,718,415 |  |
| 2022 | 995,000 | 20,350,670 |  |
| 2023 | 1,031,000 | 20,350,670 |  |
| 2024 | 1,071,000 | 21,669,048 |  |
| 2025 | 1,114,000 | 22,358,192 |  |
| 2026 | 1,161,000 | 23,067,018 |  |
| 2027 | 1,213,000 | 23,794,786 |  |
| 2028 | 1,268,000 | 24,540,844 |  |
| 2029 | 1,326,000 | 25,305,919 |  |
| 2030 | 1,389,000 | 26,090,975 |  |
