# Supplementary material for: Individual and population level costs and health-related quality of life outcomes of third-generation cephalosporin resistant bloodstream infection in Blantyre, Malawi
Source: PLOS Glob Public Health. 2023 Jun 22;3(6):e0001589. doi: 10.1371/journal.pgph.0001589 (PMC10287011; doi:10.1371/journal.pgph.0001589)
Supplement: S2 Table — (DOCX) [file pgph.0001589.s003.docx]

S2 Table

S2 Table: Observed and estimated sensitive and resistant infections

|  | Blantyre | | Malawi | | Blantyre | | Malawi | |
| --- | --- | --- | --- | --- | --- | --- | --- | --- |
| Year | *E. coli* resistant | *E. coli* grown | *E. coli* resistant | *E. coli* grown | *Klebsiella* resistant | *Klebsiella* grown | *Klebsiella* resistant | *Klebsiella* grown |
| 1998 | 115 | 185 | 2289 | 3683 | 66 | 146 | 1,314 | 2,907 |
| 1999 | 118 | 150 | 2331 | 2963 | 31 | 113 | 612 | 2,232 |
| 2000 | 83 | 116 | 1644 | 2297 | 38 | 101 | 752 | 2,000 |
| 2001 | 96 | 124 | 1909 | 2465 | 17 | 73 | 338 | 1,451 |
| 2002 | 113 | 147 | 2259 | 2939 | 20 | 81 | 400 | 1,619 |
| 2003 | 110 | 152 | 2209 | 3053 | 24 | 52 | 482 | 1,044 |
| 2004 | 92 | 133 | 1855 | 2682 | 21 | 46 | 423 | 928 |
| 2005 | 121 | 181 | 2452 | 3668 | 22 | 46 | 446 | 932 |
| 2006 | 84 | 186 | 1712 | 3791 | 18 | 52 | 367 | 1,060 |
| 2007 | 81 | 136 | 1659 | 2785 | 27 | 57 | 553 | 1,167 |
| 2008 | 81 | 116 | 1598 | 2288 | 36 | 55 | 710 | 1,085 |
| 2009 | 60 | 106 | 1189 | 2100 | 29 | 40 | 575 | 793 |
| 2010 | 64 | 96 | 1273 | 1910 | 34 | 45 | 676 | 895 |
| 2011 | 76 | 106 | 1517 | 2115 | 35 | 48 | 698 | 958 |
| 2012 | 42 | 86 | 840 | 1721 | 32 | 38 | 640 | 760 |
| 2013 | 74 | 100 | 1486 | 2007 | 33 | 55 | 662 | 1,104 |
| 2014 | 63 | 105 | 1269 | 2115 | 41 | 51 | 826 | 1,027 |
| 2015 | 65 | 118 | 1312 | 2382 | 39 | 48 | 787 | 969 |
| 2016 | 92 | 133 | 1867 | 2698 | 77 | 84 | 1,562 | 1,704 |
| 2017 |  |  | 1927 | 2786 |  |  | 1,613 | 1,760 |
| 2018 |  |  | 1988 | 2874 |  |  | 1,664 | 1,815 |
| 2019 |  |  | 2049 | 2962 |  |  | 1,715 | 1,871 |
| 2020 |  |  | 2115 | 3058 |  |  | 1,770 | 1,931 |
| 2021 |  |  | 2183 | 3156 |  |  | 1,827 | 1,993 |
| 2022 |  |  | 2253 | 3257 |  |  | 1,886 | 2,057 |
| 2023 |  |  | 2253 | 3257 |  |  | 1,886 | 2,057 |
| 2024 |  |  | 2399 | 3468 |  |  | 2,008 | 2,190 |
| 2025 |  |  | 2475 | 3578 |  |  | 2,072 | 2,260 |
| 2026 |  |  | 2554 | 3692 |  |  | 2,137 | 2,332 |
| 2027 |  |  | 2634 | 3808 |  |  | 2,205 | 2,405 |
| 2028 |  |  | 2717 | 3928 |  |  | 2,274 | 2,481 |
| 2029 |  |  | 2802 | 4050 |  |  | 2,345 | 2,558 |
| 2030 |  |  | 2889 | 4176 |  |  | 2,418 | 2,637 |
