# Supplementary material for: Individual and population level costs and health-related quality of life outcomes of third-generation cephalosporin resistant bloodstream infection in Blantyre, Malawi
Source: PLOS Glob Public Health. 2023 Jun 22;3(6):e0001589. doi: 10.1371/journal.pgph.0001589 (PMC10287011; doi:10.1371/journal.pgph.0001589)
Supplement: S3 Table — A. Annual costs (2019 US Dollars) for E. coli–Mean costs. B. Annual costs (2019 US Dollars) for E. coli– 95% Upper Credible Interval. C. Annual costs (2019 US Dollars) for E. coli– 95% Lower Credible Interval. (DOCX) [file pgph.0001589.s004.docx]

S3 Table

S3A Table: Annual costs (2019 US Dollars) for *E. coli –* Mean costs

| Year | Direct medical cost (Health Provider) | | | Societal cost | | |
| --- | --- | --- | --- | --- | --- | --- |
|  | *E. coli* 3GC-R | *E. coli* 3GC-S | All  *E. coli* | *E. coli*  3GC-R | *E. coli*  3GC-S | All  *E. coli* |
| 1998 | 724,219 | 327,451 | 1,051,670 | 1,560,067 | 645,636 | 2,205,703 |
| 1999 | 737,321 | 148,526 | 885,847 | 1,588,292 | 292,848 | 1,881,140 |
| 2000 | 519,926 | 153,551 | 673,477 | 1,119,992 | 302,757 | 1,422,749 |
| 2001 | 603,816 | 130,818 | 734,635 | 1,300,704 | 257,934 | 1,558,638 |
| 2002 | 714,586 | 159,710 | 874,296 | 1,539,317 | 314,900 | 1,854,217 |
| 2003 | 698,904 | 198,221 | 897,125 | 1,505,535 | 390,833 | 1,896,368 |
| 2004 | 586,880 | 194,277 | 781,158 | 1,264,221 | 383,057 | 1,647,277 |
| 2005 | 775,673 | 285,707 | 1,061,380 | 1,670,906 | 563,329 | 2,234,235 |
| 2006 | 541,549 | 488,467 | 1,030,016 | 1,166,571 | 963,111 | 2,129,682 |
| 2007 | 524,711 | 264,652 | 789,363 | 1,130,300 | 521,814 | 1,652,113 |
| 2008 | 505,404 | 162,218 | 667,622 | 1,088,711 | 319,845 | 1,408,556 |
| 2009 | 376,056 | 214,159 | 590,215 | 810,077 | 422,257 | 1,232,334 |
| 2010 | 402,824 | 149,611 | 552,434 | 867,738 | 294,987 | 1,162,725 |
| 2011 | 479,788 | 140,681 | 620,469 | 1,033,530 | 277,380 | 1,310,909 |
| 2012 | 265,812 | 206,850 | 472,662 | 572,596 | 407,845 | 980,441 |
| 2013 | 469,928 | 122,645 | 592,573 | 1,012,289 | 241,819 | 1,254,108 |
| 2014 | 401,474 | 198,812 | 600,286 | 864,830 | 391,998 | 1,256,827 |
| 2015 | 415,070 | 251,397 | 666,468 | 894,118 | 495,680 | 1,389,799 |
| 2016 | 590,470 | 195,466 | 785,936 | 1,271,954 | 385,400 | 1,657,354 |
| 2017 | 609,716 | 201,837 | 811,553 | 1,313,413 | 397,962 | 1,711,375 |
| 2018 | 628,962 | 208,208 | 837,170 | 1,354,872 | 410,524 | 1,765,396 |
| 2019 | 648,209 | 214,579 | 862,788 | 1,396,331 | 423,086 | 1,819,416 |
| 2020 | 669,070 | 221,485 | 890,555 | 1,441,269 | 436,702 | 1,877,971 |
| 2021 | 690,578 | 228,605 | 919,183 | 1,487,601 | 450,740 | 1,938,341 |
| 2022 | 712,721 | 235,935 | 948,656 | 1,535,300 | 465,193 | 2,000,493 |
| 2023 | 712,721 | 235,935 | 948,656 | 1,535,300 | 465,193 | 2,000,493 |
| 2024 | 758,893 | 251,220 | 1,010,113 | 1,634,761 | 495,330 | 2,130,091 |
| 2025 | 783,029 | 259,209 | 1,042,238 | 1,686,752 | 511,083 | 2,197,834 |
| 2026 | 807,853 | 267,427 | 1,075,280 | 1,740,227 | 527,286 | 2,267,513 |
| 2027 | 833,341 | 275,864 | 1,109,205 | 1,795,131 | 543,922 | 2,339,053 |
| 2028 | 859,469 | 284,514 | 1,143,983 | 1,851,416 | 560,976 | 2,412,391 |
| 2029 | 886,264 | 293,384 | 1,179,647 | 1,909,135 | 578,464 | 2,487,599 |
| 2030 | 913,758 | 302,485 | 1,216,243 | 1,968,361 | 596,410 | 2,564,771 |

*Note:*

Grey shading indicates data are based on projected BSI incidence (post 2016).

S3B Table: Annual costs (2019 US Dollars) for *E. coli –* 95% Upper Credible Interval

| Year | Direct medical cost (Health Provider) | | | Societal cost | | |
| --- | --- | --- | --- | --- | --- | --- |
|  | *E. coli* 3GC-R | *E. coli* 3GC-S | All  *E. coli* | *E. coli*  3GC-R | *E. coli*  3GC-S | All  *E. coli* |
| 1998 | 918,357 | 413,557 | 1,331,915 | 2,287,857 | 893,726 | 3,181,583 |
| 1999 | 934,972 | 187,582 | 1,122,554 | 2,329,249 | 405,377 | 2,734,626 |
| 2000 | 659,300 | 193,929 | 853,229 | 1,642,481 | 419,094 | 2,061,575 |
| 2001 | 765,679 | 165,218 | 930,897 | 1,907,498 | 357,047 | 2,264,545 |
| 2002 | 906,142 | 201,707 | 1,107,849 | 2,257,427 | 435,902 | 2,693,330 |
| 2003 | 886,256 | 250,346 | 1,136,602 | 2,207,885 | 541,014 | 2,748,899 |
| 2004 | 744,203 | 245,364 | 989,567 | 1,853,995 | 530,249 | 2,384,244 |
| 2005 | 983,604 | 360,836 | 1,344,441 | 2,450,404 | 779,792 | 3,230,196 |
| 2006 | 686,720 | 616,914 | 1,303,634 | 1,710,791 | 1,333,192 | 3,043,983 |
| 2007 | 665,368 | 334,244 | 999,612 | 1,657,598 | 722,324 | 2,379,922 |
| 2008 | 640,886 | 204,875 | 845,761 | 1,596,608 | 442,748 | 2,039,355 |
| 2009 | 476,864 | 270,474 | 747,338 | 1,187,987 | 584,512 | 1,772,500 |
| 2010 | 510,807 | 188,952 | 699,759 | 1,272,549 | 408,338 | 1,680,887 |
| 2011 | 608,403 | 177,674 | 786,077 | 1,515,684 | 383,965 | 1,899,649 |
| 2012 | 337,067 | 261,242 | 598,310 | 839,718 | 564,563 | 1,404,281 |
| 2013 | 595,899 | 154,895 | 750,795 | 1,484,534 | 334,739 | 1,819,274 |
| 2014 | 509,095 | 251,091 | 760,186 | 1,268,283 | 542,625 | 1,810,908 |
| 2015 | 526,336 | 317,505 | 843,841 | 1,311,236 | 686,149 | 1,997,384 |
| 2016 | 748,755 | 246,865 | 995,620 | 1,865,336 | 533,492 | 2,398,828 |
| 2017 | 773,160 | 254,912 | 1,028,072 | 1,926,136 | 550,881 | 2,477,017 |
| 2018 | 797,566 | 262,958 | 1,060,524 | 1,986,936 | 568,270 | 2,555,206 |
| 2019 | 821,971 | 271,005 | 1,092,976 | 2,047,736 | 585,659 | 2,633,395 |
| 2020 | 848,425 | 279,726 | 1,128,151 | 2,113,638 | 604,508 | 2,718,146 |
| 2021 | 875,699 | 288,719 | 1,164,417 | 2,181,585 | 623,940 | 2,805,525 |
| 2022 | 903,777 | 297,976 | 1,201,754 | 2,251,535 | 643,947 | 2,895,482 |
| 2023 | 903,777 | 297,976 | 1,201,754 | 2,251,535 | 643,947 | 2,895,482 |
| 2024 | 962,327 | 317,280 | 1,279,607 | 2,397,397 | 685,663 | 3,083,060 |
| 2025 | 992,932 | 327,370 | 1,320,302 | 2,473,641 | 707,470 | 3,181,111 |
| 2026 | 1,024,411 | 337,749 | 1,362,160 | 2,552,064 | 729,899 | 3,281,963 |
| 2027 | 1,056,731 | 348,405 | 1,405,137 | 2,632,582 | 752,927 | 3,385,509 |
| 2028 | 1,089,864 | 359,329 | 1,449,193 | 2,715,123 | 776,534 | 3,491,658 |
| 2029 | 1,123,841 | 370,531 | 1,494,372 | 2,799,769 | 800,743 | 3,600,512 |
| 2030 | 1,158,706 | 382,026 | 1,540,732 | 2,886,625 | 825,584 | 3,712,209 |

*Note:*

Grey shading indicates data are based on projected BSI incidence (post 2016).

S3C Table: Annual costs (2019 US Dollars) for *E. coli –* 95% Lower Credible Interval

| Year | Direct medical cost (Health Provider) | | | Societal cost | | |
| --- | --- | --- | --- | --- | --- | --- |
|  | *E. coli* 3GC-R | *E. coli* 3GC-S | All  *E. coli* | *E. coli*  3GC-R | *E. coli*  3GC-S | All  *E. coli* |
| 1998 | 530,057 | 241,345 | 771,403 | 832,254 | 397,546 | 1,229,800 |
| 1999 | 539,647 | 109,470 | 649,117 | 847,311 | 180,319 | 1,027,630 |
| 2000 | 380,535 | 113,174 | 493,709 | 597,485 | 186,421 | 783,906 |
| 2001 | 441,935 | 96,419 | 538,353 | 693,891 | 158,821 | 852,712 |
| 2002 | 523,007 | 117,713 | 640,720 | 821,185 | 193,898 | 1,015,082 |
| 2003 | 511,529 | 146,097 | 657,627 | 803,163 | 240,653 | 1,043,816 |
| 2004 | 429,539 | 143,190 | 572,729 | 674,428 | 235,865 | 910,292 |
| 2005 | 567,717 | 210,578 | 778,295 | 891,384 | 346,866 | 1,238,250 |
| 2006 | 396,361 | 360,021 | 756,381 | 622,334 | 593,029 | 1,215,363 |
| 2007 | 384,037 | 195,059 | 579,096 | 602,985 | 321,303 | 924,288 |
| 2008 | 369,907 | 119,561 | 489,468 | 580,798 | 196,942 | 777,741 |
| 2009 | 275,236 | 157,844 | 433,080 | 432,154 | 260,002 | 692,156 |
| 2010 | 294,828 | 110,269 | 405,097 | 462,915 | 181,636 | 644,551 |
| 2011 | 351,158 | 103,687 | 454,845 | 551,360 | 170,795 | 722,155 |
| 2012 | 194,548 | 152,457 | 347,005 | 305,464 | 251,128 | 556,592 |
| 2013 | 343,941 | 90,394 | 434,336 | 540,029 | 148,898 | 688,927 |
| 2014 | 293,839 | 146,533 | 440,372 | 461,363 | 241,370 | 702,733 |
| 2015 | 303,791 | 185,290 | 489,081 | 476,988 | 305,212 | 782,200 |
| 2016 | 432,166 | 144,066 | 576,233 | 678,553 | 237,307 | 915,861 |
| 2017 | 446,253 | 148,762 | 595,015 | 700,671 | 245,042 | 945,713 |
| 2018 | 460,339 | 153,458 | 613,797 | 722,788 | 252,777 | 975,565 |
| 2019 | 474,425 | 158,154 | 632,579 | 744,905 | 260,512 | 1,005,417 |
| 2020 | 489,694 | 163,244 | 652,937 | 768,878 | 268,896 | 1,037,775 |
| 2021 | 505,436 | 168,491 | 673,927 | 793,595 | 277,540 | 1,071,136 |
| 2022 | 521,642 | 173,894 | 695,536 | 819,041 | 286,439 | 1,105,481 |
| 2023 | 521,642 | 173,894 | 695,536 | 819,041 | 286,439 | 1,105,481 |
| 2024 | 555,436 | 185,159 | 740,595 | 872,101 | 304,996 | 1,177,097 |
| 2025 | 573,100 | 191,048 | 764,148 | 899,837 | 314,696 | 1,214,533 |
| 2026 | 591,270 | 197,105 | 788,374 | 928,364 | 324,673 | 1,253,037 |
| 2027 | 609,924 | 203,323 | 813,248 | 957,655 | 334,916 | 1,292,571 |
| 2028 | 629,048 | 209,698 | 838,746 | 987,681 | 345,417 | 1,333,098 |
| 2029 | 648,659 | 216,236 | 864,894 | 1,018,472 | 356,186 | 1,374,658 |
| 2030 | 668,782 | 222,944 | 891,726 | 1,050,068 | 367,235 | 1,417,303 |

*Note:*

Grey shading indicates data are based on projected BSI incidence (post 2016).
