# Supplementary material for: Individual and population level costs and health-related quality of life outcomes of third-generation cephalosporin resistant bloodstream infection in Blantyre, Malawi
Source: PLOS Glob Public Health. 2023 Jun 22;3(6):e0001589. doi: 10.1371/journal.pgph.0001589 (PMC10287011; doi:10.1371/journal.pgph.0001589)
Supplement: S4 Table — A. Annual costs (2019 US Dollars) for Klebsiella spp.–Mean costs. B. Annual costs (2019 US Dollars) for Klebsiella spp. - 95% Upper Credible Interval. C. Annual costs (2019 US Dollars) for Klebsiella spp. - 95% Lower Credible Interval. (DOCX) [file pgph.0001589.s005.docx]

S4 Table

S4A Table: Annual costs (2019 US Dollars) for *Klebsiella* spp. – Mean costs

| Year | Direct medical cost (Health Provider) | | | Societal cost | | |
| --- | --- | --- | --- | --- | --- | --- |
|  | *Klebsiella* 3GC-R | *Klebsiella* 3GC-S | All *Klebsiella* | *Klebsiella* 3GC-R | *Klebsiella* 3GC-S | All *Klebsiella* |
| 1998 | 503,775 | 331,876 | 835,651 | 938,623 | 613,216 | 1,551,839 |
| 1999 | 234,778 | 337,523 | 572,301 | 437,433 | 623,649 | 1,061,082 |
| 2000 | 288,514 | 259,967 | 548,481 | 537,554 | 480,347 | 1,017,901 |
| 2001 | 129,599 | 232,026 | 361,625 | 241,467 | 428,720 | 670,187 |
| 2002 | 153,295 | 254,109 | 407,404 | 285,615 | 469,524 | 755,139 |
| 2003 | 184,823 | 117,192 | 302,015 | 344,359 | 216,538 | 560,897 |
| 2004 | 162,368 | 105,055 | 267,423 | 302,521 | 194,112 | 496,634 |
| 2005 | 170,937 | 101,349 | 272,286 | 318,486 | 187,265 | 505,751 |
| 2006 | 140,654 | 144,395 | 285,049 | 262,063 | 266,802 | 528,865 |
| 2007 | 211,992 | 128,018 | 340,010 | 394,979 | 236,542 | 631,521 |
| 2008 | 272,256 | 78,095 | 350,351 | 507,261 | 144,298 | 651,559 |
| 2009 | 220,303 | 45,416 | 265,719 | 410,464 | 83,916 | 494,380 |
| 2010 | 259,379 | 45,608 | 304,987 | 483,270 | 84,271 | 567,541 |
| 2011 | 267,809 | 54,062 | 321,871 | 498,975 | 99,892 | 598,867 |
| 2012 | 245,469 | 25,014 | 270,483 | 457,352 | 46,220 | 503,572 |
| 2013 | 254,000 | 92,031 | 346,032 | 473,248 | 170,049 | 643,296 |
| 2014 | 316,680 | 41,979 | 358,659 | 590,032 | 77,565 | 667,597 |
| 2015 | 301,852 | 37,859 | 339,710 | 562,403 | 69,952 | 632,356 |
| 2016 | 598,993 | 29,595 | 628,588 | 1,116,030 | 54,684 | 1,170,714 |
| 2017 | 618,517 | 30,560 | 649,076 | 1,152,407 | 56,466 | 1,208,873 |
| 2018 | 638,041 | 31,525 | 669,565 | 1,188,784 | 58,249 | 1,247,032 |
| 2019 | 657,564 | 32,489 | 690,054 | 1,225,160 | 60,031 | 1,285,191 |
| 2020 | 678,727 | 33,535 | 712,262 | 1,264,589 | 61,963 | 1,326,553 |
| 2021 | 700,546 | 34,613 | 735,159 | 1,305,242 | 63,955 | 1,369,197 |
| 2022 | 723,008 | 35,723 | 758,731 | 1,347,093 | 66,006 | 1,413,099 |
| 2023 | 723,008 | 35,723 | 758,731 | 1,347,093 | 66,006 | 1,413,099 |
| 2024 | 769,847 | 38,037 | 807,884 | 1,434,362 | 70,282 | 1,504,644 |
| 2025 | 794,330 | 39,247 | 833,577 | 1,479,979 | 72,517 | 1,552,496 |
| 2026 | 819,513 | 40,491 | 860,004 | 1,526,899 | 74,816 | 1,601,715 |
| 2027 | 845,369 | 41,768 | 887,137 | 1,575,073 | 77,176 | 1,652,250 |
| 2028 | 871,874 | 43,078 | 914,952 | 1,624,458 | 79,596 | 1,704,054 |
| 2029 | 899,056 | 44,421 | 943,477 | 1,675,101 | 82,078 | 1,757,179 |
| 2030 | 926,947 | 45,799 | 972,746 | 1,727,067 | 84,624 | 1,811,691 |

*Note:*

Grey shading indicates data are based on projected BSI incidence (post 2016).

S4B Table: Annual costs (2019 US Dollars) for *Klebsiella* spp. - 95% Upper Credible Interval

| Year | Direct medical cost (Health Provider) | | | Societal cost | | |
| --- | --- | --- | --- | --- | --- | --- |
|  | *Klebsiella* 3GC-R | *Klebsiella* 3GC-S | All *Klebsiella* | *Klebsiella* 3GC-R | *Klebsiella* 3GC-S | All *Klebsiella* |
| 1998 | 698,219 | 507,993 | 1,206,212 | 1,466,770 | 1,063,875 | 2,530,646 |
| 1999 | 325,396 | 516,636 | 842,032 | 683,570 | 1,081,976 | 1,765,545 |
| 2000 | 399,873 | 397,923 | 797,797 | 840,027 | 833,360 | 1,673,386 |
| 2001 | 179,621 | 355,155 | 534,776 | 377,337 | 743,790 | 1,121,127 |
| 2002 | 212,462 | 388,957 | 601,419 | 446,326 | 814,582 | 1,260,908 |
| 2003 | 256,160 | 179,382 | 435,542 | 538,124 | 375,674 | 913,798 |
| 2004 | 225,038 | 160,804 | 385,842 | 472,745 | 336,768 | 809,513 |
| 2005 | 236,914 | 155,132 | 392,046 | 497,694 | 324,888 | 822,581 |
| 2006 | 194,942 | 221,021 | 415,963 | 409,522 | 462,878 | 872,400 |
| 2007 | 293,815 | 195,953 | 489,768 | 617,227 | 410,379 | 1,027,606 |
| 2008 | 377,339 | 119,537 | 496,877 | 792,689 | 250,344 | 1,043,033 |
| 2009 | 305,334 | 69,517 | 374,851 | 641,425 | 145,587 | 787,012 |
| 2010 | 359,493 | 69,811 | 429,303 | 755,197 | 146,203 | 901,401 |
| 2011 | 371,176 | 82,751 | 453,927 | 779,741 | 173,304 | 953,044 |
| 2012 | 340,213 | 38,289 | 378,502 | 714,696 | 80,187 | 794,883 |
| 2013 | 352,037 | 140,870 | 492,907 | 739,536 | 295,020 | 1,034,556 |
| 2014 | 438,910 | 64,256 | 503,166 | 922,033 | 134,569 | 1,056,602 |
| 2015 | 418,358 | 57,949 | 476,307 | 878,859 | 121,361 | 1,000,220 |
| 2016 | 830,188 | 45,301 | 875,488 | 1,744,002 | 94,872 | 1,838,874 |
| 2017 | 857,248 | 46,777 | 904,025 | 1,800,847 | 97,964 | 1,898,812 |
| 2018 | 884,307 | 48,254 | 932,561 | 1,857,693 | 101,056 | 1,958,749 |
| 2019 | 911,367 | 49,730 | 961,097 | 1,914,538 | 104,149 | 2,018,687 |
| 2020 | 940,697 | 51,331 | 992,028 | 1,976,153 | 107,501 | 2,083,654 |
| 2021 | 970,938 | 52,981 | 1,023,919 | 2,039,680 | 110,956 | 2,150,637 |
| 2022 | 1,002,070 | 54,680 | 1,056,750 | 2,105,081 | 114,514 | 2,219,595 |
| 2023 | 1,002,070 | 54,680 | 1,056,750 | 2,105,081 | 114,514 | 2,219,595 |
| 2024 | 1,066,987 | 58,222 | 1,125,209 | 2,241,454 | 121,933 | 2,363,387 |
| 2025 | 1,100,921 | 60,074 | 1,160,994 | 2,312,740 | 125,810 | 2,438,550 |
| 2026 | 1,135,824 | 61,978 | 1,197,802 | 2,386,061 | 129,799 | 2,515,860 |
| 2027 | 1,171,659 | 63,934 | 1,235,593 | 2,461,342 | 133,894 | 2,595,236 |
| 2028 | 1,208,395 | 65,938 | 1,274,333 | 2,538,514 | 138,092 | 2,676,606 |
| 2029 | 1,246,067 | 67,994 | 1,314,061 | 2,617,654 | 142,397 | 2,760,051 |
| 2030 | 1,284,724 | 70,103 | 1,354,827 | 2,698,860 | 146,815 | 2,845,675 |

*Note:*

Grey shading indicates data are based on projected BSI incidence (post 2016).

S4C Table Annual costs (2019 US Dollars) for *Klebsiella* spp. - 95% Lower Credible Interval

| Year | Direct medical cost (Health Provider) | | | Societal cost | | |
| --- | --- | --- | --- | --- | --- | --- |
|  | *Klebsiella* 3GC-R | *Klebsiella* 3GC-S | All *Klebsiella* | *Klebsiella* 3GC-R | *Klebsiella* 3GC-S | All *Klebsiella* |
| 1998 | 309,344 | 155,773 | 465,117 | 410,488 | 162,573 | 573,061 |
| 1999 | 144,166 | 158,423 | 302,589 | 191,303 | 165,339 | 356,642 |
| 2000 | 177,163 | 122,021 | 299,183 | 235,089 | 127,347 | 362,436 |
| 2001 | 79,581 | 108,906 | 188,487 | 105,601 | 113,660 | 219,261 |
| 2002 | 94,131 | 119,271 | 213,402 | 124,908 | 124,478 | 249,386 |
| 2003 | 113,491 | 55,006 | 168,497 | 150,598 | 57,408 | 208,006 |
| 2004 | 99,703 | 49,310 | 149,012 | 132,302 | 51,462 | 183,764 |
| 2005 | 104,964 | 47,570 | 152,534 | 139,284 | 49,647 | 188,931 |
| 2006 | 86,369 | 67,775 | 154,143 | 114,608 | 70,733 | 185,341 |
| 2007 | 130,174 | 60,088 | 190,262 | 172,736 | 62,711 | 235,447 |
| 2008 | 167,179 | 36,655 | 203,835 | 221,841 | 38,256 | 260,096 |
| 2009 | 135,278 | 21,317 | 156,594 | 179,508 | 22,247 | 201,756 |
| 2010 | 159,272 | 21,407 | 180,679 | 211,348 | 22,342 | 233,690 |
| 2011 | 164,449 | 25,375 | 189,824 | 218,217 | 26,483 | 244,700 |
| 2012 | 150,731 | 11,741 | 162,472 | 200,014 | 12,254 | 212,267 |
| 2013 | 155,969 | 43,197 | 199,166 | 206,966 | 45,083 | 252,048 |
| 2014 | 194,458 | 19,704 | 214,162 | 258,039 | 20,564 | 278,603 |
| 2015 | 185,353 | 17,770 | 203,122 | 245,956 | 18,545 | 264,501 |
| 2016 | 367,813 | 13,891 | 381,704 | 488,074 | 14,498 | 502,571 |
| 2017 | 379,802 | 14,344 | 394,146 | 503,982 | 14,970 | 518,953 |
| 2018 | 391,790 | 14,797 | 406,587 | 519,891 | 15,443 | 535,334 |
| 2019 | 403,779 | 15,249 | 419,029 | 535,800 | 15,915 | 551,715 |
| 2020 | 416,774 | 15,740 | 432,514 | 553,043 | 16,427 | 569,471 |
| 2021 | 430,172 | 16,246 | 446,418 | 570,822 | 16,955 | 587,777 |
| 2022 | 443,965 | 16,767 | 460,732 | 589,125 | 17,499 | 606,624 |
| 2023 | 443,965 | 16,767 | 460,732 | 589,125 | 17,499 | 606,624 |
| 2024 | 472,726 | 17,853 | 490,580 | 627,290 | 18,633 | 645,923 |
| 2025 | 487,761 | 18,421 | 506,182 | 647,240 | 19,225 | 666,465 |
| 2026 | 503,224 | 19,005 | 522,229 | 667,759 | 19,835 | 687,594 |
| 2027 | 519,101 | 19,605 | 538,706 | 688,827 | 20,461 | 709,288 |
| 2028 | 535,377 | 20,219 | 555,596 | 710,425 | 21,102 | 731,527 |
| 2029 | 552,067 | 20,850 | 572,917 | 732,573 | 21,760 | 754,333 |
| 2030 | 569,194 | 21,497 | 590,691 | 755,299 | 22,435 | 777,734 |

*Note:*

Grey shading indicates data are based on projected BSI incidence (post 2016).
