# Supplementary material for: Individual and population level costs and health-related quality of life outcomes of third-generation cephalosporin resistant bloodstream infection in Blantyre, Malawi
Source: PLOS Glob Public Health. 2023 Jun 22;3(6):e0001589. doi: 10.1371/journal.pgph.0001589 (PMC10287011; doi:10.1371/journal.pgph.0001589)
Supplement: S5 Table — A. Annual QALYs lost due to E. coli and Klebsiella spp.—Mean QALYs. B. Annual QALYs lost due to E. coli and Klebsiella spp.– 95% Upper Credible Interval QALYs. C. Annual QALYs lost due to E. coli and Klebsiella spp.–Lower 95% Credible Interval QALYs. (DOCX) [file pgph.0001589.s006.docx]

S5 Table

S5A Table: Annual QALYs lost due to *E. coli* and *Klebsiella* spp. - Mean QALYs

| Year | Total QALYs Lost | | | Total QALYs Lost | | |
| --- | --- | --- | --- | --- | --- | --- |
|  | *E. coli* Resistant | *E. coli* Sensitive | All *E. coli* | *Klebsiella* Resistant | *Klebsiella* Sensitive | All *Klebsiella* |
| 1998 | 56,516 | 22,843 | 79,358 | 28,508 | 40,352 | 68,860 |
| 1999 | 57,538 | 10,361 | 67,899 | 13,286 | 41,039 | 54,325 |
| 2000 | 40,573 | 10,712 | 51,285 | 16,327 | 31,609 | 47,936 |
| 2001 | 47,120 | 9,126 | 56,246 | 7,334 | 28,212 | 35,545 |
| 2002 | 55,764 | 11,141 | 66,905 | 8,675 | 30,897 | 39,571 |
| 2003 | 54,540 | 13,828 | 68,368 | 10,459 | 14,249 | 24,708 |
| 2004 | 45,798 | 13,553 | 59,351 | 9,188 | 12,773 | 21,962 |
| 2005 | 60,531 | 19,931 | 80,462 | 9,673 | 12,323 | 21,996 |
| 2006 | 42,261 | 34,075 | 76,336 | 7,959 | 17,557 | 25,516 |
| 2007 | 40,947 | 18,462 | 59,408 | 11,996 | 15,565 | 27,562 |
| 2008 | 39,440 | 11,316 | 50,756 | 15,407 | 9,495 | 24,902 |
| 2009 | 29,346 | 14,939 | 44,286 | 12,467 | 5,522 | 17,989 |
| 2010 | 31,435 | 10,437 | 41,872 | 14,678 | 5,545 | 20,223 |
| 2011 | 37,441 | 9,814 | 47,255 | 15,155 | 6,573 | 21,728 |
| 2012 | 20,743 | 14,430 | 35,173 | 13,891 | 3,041 | 16,932 |
| 2013 | 36,672 | 8,556 | 45,227 | 14,374 | 11,190 | 25,564 |
| 2014 | 31,330 | 13,869 | 45,199 | 17,921 | 5,104 | 23,025 |
| 2015 | 32,391 | 17,537 | 49,928 | 17,082 | 4,603 | 21,685 |
| 2016 | 46,078 | 13,635 | 59,714 | 33,897 | 3,598 | 37,495 |
| 2017 | 47,580 | 14,080 | 61,660 | 35,001 | 3,716 | 38,717 |
| 2018 | 49,082 | 14,524 | 63,606 | 36,106 | 3,833 | 39,939 |
| 2019 | 50,584 | 14,969 | 65,553 | 37,211 | 3,950 | 41,161 |
| 2020 | 52,212 | 15,451 | 67,663 | 38,409 | 4,077 | 42,486 |
| 2021 | 53,890 | 15,947 | 69,838 | 39,643 | 4,209 | 43,852 |
| 2022 | 55,618 | 16,459 | 72,077 | 40,914 | 4,343 | 45,258 |
| 2023 | 55,618 | 16,459 | 72,077 | 40,914 | 4,343 | 45,258 |
| 2024 | 59,221 | 17,525 | 76,746 | 43,565 | 4,625 | 48,190 |
| 2025 | 61,105 | 18,082 | 79,187 | 44,951 | 4,772 | 49,722 |
| 2026 | 63,042 | 18,655 | 81,698 | 46,376 | 4,923 | 51,299 |
| 2027 | 65,031 | 19,244 | 84,275 | 47,839 | 5,079 | 52,917 |
| 2028 | 67,070 | 19,847 | 86,917 | 49,339 | 5,238 | 54,576 |
| 2029 | 69,161 | 20,466 | 89,627 | 50,877 | 5,401 | 56,278 |
| 2030 | 71,307 | 21,101 | 92,408 | 52,455 | 5,569 | 58,024 |

S5B Table: Annual QALYs lost due to *E. coli* and *Klebsiella* spp. – 95% Upper Credible Interval QALYs

| Year | Total QALYs Lost | | | Total QALYs Lost | | |
| --- | --- | --- | --- | --- | --- | --- |
|  | *E. coli* Resistant | *E. coli* Sensitive | All *E. coli* | *Klebsiella* Resistant | *Klebsiella* Sensitive | All *Klebsiella* |
| 1998 | 56,531 | 22,851 | 79,382 | 28,522 | 40,363 | 68,885 |
| 1999 | 57,553 | 10,365 | 67,918 | 13,292 | 41,050 | 54,342 |
| 2000 | 40,584 | 10,716 | 51,300 | 16,335 | 31,617 | 47,952 |
| 2001 | 47,132 | 9,129 | 56,262 | 7,337 | 28,219 | 35,556 |
| 2002 | 55,779 | 11,145 | 66,924 | 8,679 | 30,905 | 39,584 |
| 2003 | 54,555 | 13,833 | 68,388 | 10,464 | 14,253 | 24,717 |
| 2004 | 45,810 | 13,558 | 59,368 | 9,193 | 12,777 | 21,969 |
| 2005 | 60,547 | 19,938 | 80,485 | 9,678 | 12,326 | 22,004 |
| 2006 | 42,272 | 34,088 | 76,360 | 7,963 | 17,561 | 25,525 |
| 2007 | 40,958 | 18,469 | 59,426 | 12,002 | 15,570 | 27,572 |
| 2008 | 39,451 | 11,320 | 50,771 | 15,414 | 9,498 | 24,912 |
| 2009 | 29,354 | 14,945 | 44,299 | 12,473 | 5,524 | 17,996 |
| 2010 | 31,443 | 10,441 | 41,884 | 14,685 | 5,547 | 20,232 |
| 2011 | 37,451 | 9,817 | 47,269 | 15,162 | 6,575 | 21,737 |
| 2012 | 20,749 | 14,435 | 35,184 | 13,897 | 3,042 | 16,940 |
| 2013 | 36,681 | 8,559 | 45,240 | 14,381 | 11,193 | 25,573 |
| 2014 | 31,338 | 13,874 | 45,212 | 17,929 | 5,105 | 23,035 |
| 2015 | 32,399 | 17,544 | 49,943 | 17,090 | 4,604 | 21,694 |
| 2016 | 46,091 | 13,641 | 59,731 | 33,913 | 3,599 | 37,512 |
| 2017 | 47,593 | 14,085 | 61,678 | 35,018 | 3,717 | 38,735 |
| 2018 | 49,095 | 14,530 | 63,625 | 36,123 | 3,834 | 39,957 |
| 2019 | 50,597 | 14,975 | 65,572 | 37,229 | 3,951 | 41,180 |
| 2020 | 52,226 | 15,456 | 67,682 | 38,427 | 4,079 | 42,505 |
| 2021 | 53,905 | 15,953 | 69,858 | 39,662 | 4,210 | 43,872 |
| 2022 | 55,633 | 16,465 | 72,098 | 40,934 | 4,345 | 45,279 |
| 2023 | 55,633 | 16,465 | 72,098 | 40,934 | 4,345 | 45,279 |
| 2024 | 59,237 | 17,532 | 76,769 | 43,586 | 4,626 | 48,212 |
| 2025 | 61,121 | 18,089 | 79,210 | 44,972 | 4,773 | 49,745 |
| 2026 | 63,059 | 18,663 | 81,722 | 46,398 | 4,925 | 51,322 |
| 2027 | 65,048 | 19,251 | 84,300 | 47,862 | 5,080 | 52,941 |
| 2028 | 67,088 | 19,855 | 86,943 | 49,362 | 5,239 | 54,601 |
| 2029 | 69,179 | 20,474 | 89,653 | 50,901 | 5,402 | 56,304 |
| 2030 | 71,326 | 21,109 | 92,435 | 52,480 | 5,570 | 58,050 |

S5C Table: Annual QALYs lost due to *E. coli* and *Klebsiella* spp. – Lower 95% Credible Interval QALYs

| Year | Total QALYs Lost | | | Total QALYs Lost | | |
| --- | --- | --- | --- | --- | --- | --- |
|  | *E. coli* Resistant | *E. coli* Sensitive | All *E. coli* | *Klebsiella* Resistant | *Klebsiella* Sensitive | All *Klebsiella* |
| 1998 | 56,501 | 22,834 | 79,334 | 28,495 | 40,341 | 68,836 |
| 1999 | 57,523 | 10,357 | 67,880 | 13,280 | 41,028 | 54,307 |
| 2000 | 40,562 | 10,707 | 51,270 | 16,319 | 31,600 | 47,919 |
| 2001 | 47,107 | 9,122 | 56,229 | 7,330 | 28,204 | 35,534 |
| 2002 | 55,749 | 11,137 | 66,886 | 8,671 | 30,888 | 39,559 |
| 2003 | 54,526 | 13,822 | 68,348 | 10,454 | 14,245 | 24,699 |
| 2004 | 45,786 | 13,547 | 59,333 | 9,184 | 12,770 | 21,954 |
| 2005 | 60,515 | 19,923 | 80,438 | 9,669 | 12,320 | 21,988 |
| 2006 | 42,249 | 34,062 | 76,311 | 7,956 | 17,552 | 25,508 |
| 2007 | 40,936 | 18,455 | 59,390 | 11,991 | 15,561 | 27,552 |
| 2008 | 39,430 | 11,312 | 50,741 | 15,399 | 9,493 | 24,892 |
| 2009 | 29,338 | 14,934 | 44,272 | 12,461 | 5,521 | 17,981 |
| 2010 | 31,427 | 10,433 | 41,859 | 14,671 | 5,544 | 20,215 |
| 2011 | 37,431 | 9,810 | 47,241 | 15,148 | 6,572 | 21,719 |
| 2012 | 20,738 | 14,424 | 35,162 | 13,884 | 3,041 | 16,925 |
| 2013 | 36,662 | 8,552 | 45,214 | 14,367 | 11,187 | 25,554 |
| 2014 | 31,321 | 13,864 | 45,185 | 17,912 | 5,103 | 23,015 |
| 2015 | 32,382 | 17,530 | 49,913 | 17,073 | 4,602 | 21,675 |
| 2016 | 46,066 | 13,630 | 59,696 | 33,880 | 3,597 | 37,478 |
| 2017 | 47,568 | 14,075 | 61,642 | 34,985 | 3,715 | 38,699 |
| 2018 | 49,069 | 14,519 | 63,588 | 36,089 | 3,832 | 39,921 |
| 2019 | 50,571 | 14,963 | 65,534 | 37,193 | 3,949 | 41,143 |
| 2020 | 52,198 | 15,445 | 67,643 | 38,390 | 4,076 | 42,467 |
| 2021 | 53,876 | 15,941 | 69,817 | 39,624 | 4,207 | 43,832 |
| 2022 | 55,604 | 16,452 | 72,056 | 40,895 | 4,342 | 45,237 |
| 2023 | 55,604 | 16,452 | 72,056 | 40,895 | 4,342 | 45,237 |
| 2024 | 59,206 | 17,518 | 76,724 | 43,544 | 4,624 | 48,168 |
| 2025 | 61,089 | 18,075 | 79,164 | 44,929 | 4,771 | 49,700 |
| 2026 | 63,025 | 18,648 | 81,674 | 46,354 | 4,922 | 51,275 |
| 2027 | 65,014 | 19,237 | 84,250 | 47,816 | 5,077 | 52,893 |
| 2028 | 67,052 | 19,840 | 86,892 | 49,315 | 5,236 | 54,552 |
| 2029 | 69,143 | 20,458 | 89,601 | 50,853 | 5,400 | 56,252 |
| 2030 | 71,288 | 21,093 | 92,381 | 52,430 | 5,567 | 57,997 |
