# Supplementary material for: Individual and population level costs and health-related quality of life outcomes of third-generation cephalosporin resistant bloodstream infection in Blantyre, Malawi
Source: PLOS Glob Public Health. 2023 Jun 22;3(6):e0001589. doi: 10.1371/journal.pgph.0001589 (PMC10287011; doi:10.1371/journal.pgph.0001589)
Supplement: Attachment — Submitted filename: Response to Reviewers_hm_nf_rl.docx [file pgph.0001589.s007.docx]

20^th^ January 2023

Dear Editor

**Manuscript ID: PGPH-D-22-01156R1**

**Manuscript Title: Individual and population level costs and health-related quality of life outcomes of third-generation cephalosporin resistant bloodstream infections in Malawi.**

We are grateful for the helpful comments provided by the reviewers and have revised the manuscript accordingly. Full point-by-point responses to reviewers are provided below.

Yours sincerely,

Dr Rebecca Lester (on behalf of the authors)

**Response to Reviewer 1:**

1. *The title refers to Malawi, but I noticed you performed your research at the Queen Elizabeth Central Hospital (QECH) in Blantyre, so I don’t know if one hospital qualifies as a whole country (but I might be wrong). Likely, it is worth rewording the title and referring to the city and country (if the hospital is the largest in the town). Please also state whether it is a primary/secondary/tertiary care hospital or public/private throughout the introduction.*

We have re-worded the title as follows:

Individual and population level costs and health-related quality of life outcomes of third-generation cephalosporin resistant bloodstream infections in Blantyre, Malawi.

We have reworded this as follows:

QECH is a 1300 bedded teaching public hospital which provides free inpatient secondary care to Blantyre and tertiary care to surrounding districts.

2. *I think readers are not entirely familiar with the specific studied city in Malawi. I recommend you provide some context about the city and how it is compared to the capital city in Malawi or the percentage of hospital patients attended compared to the whole country.*

We have amended the fist paragraph of Methods as follows, to provide more detail on the study setting:

This was a sub-study of a prospective longitudinal cohort of patients at QECH who had bloodstream Enterobacterales, protocol detailed at [4]. Malawi is a low-income country with low healthcare expenditure (~ 3% GDP) and an estimated population of 17.5 million people Blantyre is the second city and commercial capital, with a population of 800,264. QECH is a 1300 bedded teaching public hospital which provides free inpatient secondary care to Blantyre and tertiary care to surrounding districts

3. *How are the 3GC-R and 3GC-S infections defined based on MICs? Which thresholds did you use; CLSI, EUCAST?*

We have expanded on the methods as follows:

An overview of the data collection methods is shown in Figure 1 and the CRFs used are included as extended data in the published protocol [4]. The detailed laboratory methods are described elsewhere, but briefly, blood cultures were processed at the ISO accredited, quality assured diagnostic laboratory at MLW (UK National External Quality Assessment Service. Antimicrobial susceptibility (AST) was established using the disc diffusion method and EUCAST breakpoints (EUCAST version 7.0). 3GC-R was defined as resistance to one or both of cefpodoxime or ceftriaxone and was confirmed using the species dependent combination disc method

4. *Why did you construct a parallel model including HIV infection? It is worth an in-depth explanation rather than just citing the paper. Likely, this will give us more context about the disease epidemiology of the targeted population. Are other specific features such as a high HIV prevalence embedded in the population?*

We have added to the methods to highlight the high HIV prevalence amongst medical inpatients admitted to QECH. We included a parallel model with HIV infection to account for additional healthcare resources utilised to manage their HIV infection during admission, and additional detriment to individuals’ HRQoL from HIV. We also discuss the findings of HIV-infection only having a marginal effect on costs and HRQoL outcomes in the discussion.

5. *I have seen that you modelled and projected the estimates using the chosen hospital as the reference. First, you should state that you projected the costs using the reference hospital in the abstract.*

We have added to the abstract to highlight we used cost and microbiology surveillance data from Queen Elizabeth Central Hospital to model estimates for Malawi.

6. *I noticed you described 154 patients on line 205 (results), but it is not equivalent to what is illustrated in Figure 1. Would you mind adding the exact sample size throughout the methods section? Does that differ by the outcome? Is there any missing data, how Is it handled and was it at random? Was the study fully randomised? If missing data were due to death, I presume the costs are highly underestimated. How would you handle this?*

We agree with the reviewer around the issue of missing data. We have therefore redone our analysis and model projections using estimates derived from imputed datasets. In brief, we undertook Multiple Imputation using chained equations to impute missing cost and HRQoL estimates. We imputed 10 datasets and the results were combined using Rubin’s rules. All estimates now presented in the manuscript are from the imputed datasets and include all 154 participants. We have highlighted in Figure 1 and in the Table headings the analysis was undertaken on the full 154 sample.

7. *Table 1; would you mind inserting all the abbreviations fully described in Table 1’s notes? This might include ART, HIV, 3GC-S and R, CI, and IQR. Also, you have enough space to write Escherichia coli instead of E. coli. Could you please state the total number of observations next to Table 1’s title (e.g., {N=154})?*

This change has been made

8. *Tables 2-7; state the total number of obs in the title. Refer to the acronyms/abbreviations in full in notes (this might include 3GC, SE, CR, etc.). Coef should be written as “Coeff.”. The “Ref” term should be written with a dot by the end and describe it fully in notes.*

We undertook multiple imputation and have made changes to Tables 2-7 to highlight the total sample used for analysis was 154 participants. We have also indicated the reference category in the footnotes. Acronyms and abbreviations have been explained in full in the notes of these tables

9. *Table 5. Could you pls replicate the analyses for total indirect costs? Would you mind presenting the p-values? You might want to add rows to refer to the other variables included in the analysis (you can put a tick box to show what is included or not, and present the full results in the supplementary material).*

We have replicated the analyses for total indirect costs in Table 5. Tables 5 and 7 highlight the additional variables included in the multivariable models. As is standard practice for health economics studies of this nature, we have not included p-values, as we are making estimates, and not inferences, around the costs, and HRQoL detriments from sensitive and resistant blood stream infections.

10. *What were multivariable analyses employed? Linear methods using linear regressions? Did you test the distributions? Skewness, multicollinearity of independent variables, homoscedasticity? Did you use robust SE? How the values for the time series were predicted (till 2030)?*

We have added to the methods around the approach we used to undertake the multivariable analysis and choice of estimators. We have added in the footnotes of Tables 5 and 7 the estimators used in the multivariable analysis. Briefly, for the cost estimates we use generalised linear models, and use a range of diagnostics including the Park test, linktest, Akaike information criterion, and visually inspect the plots of the deviance residuals to determine the optimal choice for the link function and the distributional family. For EQ-5D utility data we used a range of common estimators and compare the mean squared error and mean absolute error statistics between the observed EQ-5D utility score and the estimated scores for the whole sample, and for sub-groups of the sample based on observed EQ-5D utility scores to determine the choice of estimator. We have included references to justify this approach.

11. *I wonder if using the Zimbabwean tariff for the EQ-5D impacts the estimates, why you chose Zimbabwe and not any other, and how that might be linked to Malawi. What is the rationale for using UK and Zimbabwe’s tariffs? Likely, it is worth having a word in the limitation’s subsection.*

We have added to the limitations to highlight the use of the Zimbabwean tariff. We used the UK tariff in our sensitivity analysis as it yields lower EQ-5D utility scores than the Zimbabwean tariff for the same health states (responses to the descriptive component of EQ-5D-3L).

12. *Did you inflate and transform to 2019 USD the costs you mentioned in the discussion from other articles?*

We have added to the text to highlight the original price year (e.g. Line) for costs referenced from other articles.

13. *The abstract should be rewritten, accounting for some of the comments I have made throughout. For instance, define the multivariable methods used; you mentioned the mean health provider costs in results but did not define it in methods, etc. Also, state the currency throughout the abstract (2019 USD).*

We have made changes in the abstract to account for all issues raised.

14. *Sensitivity analyses. I would expect to see variations in cost estimates, given how the data were reported (indexes used) or the targeted population. For instance, you did not include children or ppl aged <18 years. How might the estimates vary if we can account for that? You used the Zimbabwean and UK tariffs for EQ-5D; would it change if a Malawian index were constructed? I know that it's not available everywhere, but how might it affect the estimates?*

We have highlighted in the discussion that our findings are limited because they do not include children) and because we used the Zimbabwean tariff. We used the UK tariff to derive EQ-5D utility scores in our sensitivity analysis as it yields lower EQ-5D utility scores than the Zimbabwean tariff for the same health states.

15. *Spell out the LMICs abbreviation once first mentioned.*

This change has been made

16. *Spell out TB once first mentioned.*

This change has been made

17. *I think the sentence highlighting the software used should be moved to the last part of the statistical analyses subsection.*

This change has been made

18. *Line 83.. it says “to informed” should it be to inform?*

This change has been made

19. *HIV and ART should be spelt out once first mentioned*

This change has been made

20. *IQR, spell this out.*

This change has been made

21. *Table 2; the “n” should be written in capital letters to be consistent with the rest.*

This change has been made

22. *I will use a subtitle in the methods section for the societal costs. Societal costs= direct and indirect medical and non-medical costs; so then you describe them all. I did not realise they were calculated as the sum till I got to the statistical analyses’ subsection.*

This change has been made

23. *Why do you use 2019 USD and international dollars?*

There was a typo and all costs we estimated are in 2019 US Dollars. This has been amended.

24. *Could you pls expand the labels and legends in Figures 2-4 and make the lines thicker? Figure 4 should be similar to the previous ones (legends on top favour the reading, no need to repeat the legends in both panels). Also, A and B are bolded in Figures2-3 and not in Figure 4. It should be great to make them consistent.*

These changes have been made

25. *Could you please present the 95% CI for the predicted values in Figures 2-4.*

We have included the 95% CR for all our projected costs and QALYs lost (Lines 855-899).

**Response to Reviewer 2:** Even the manuscript is technically sound and it represents an important additional information on cost impact of AMR in a Low Income Country, some major revision should been made:

1. *There is not any discussion on EQ-5D limitations use in the african context. It must be included in the methodology section and in the discussion one.*

We have added to the limitations to highlight the use of the Zimbabwean tariff. We used the UK tariff in our sensitivity analysis as it yields lower EQ-5D utility scores than the Zimbabwean tariff for the same health states (responses to the descriptive component of EQ-5D-3L).

2. *The societal cost in terms of out of pocket costs and loss of income seem to be hidden in the findings. Those are not discussed at all. It would be relevant to be included some comments around them, given the Country context.*

We have added the following paragraph in the discussion:

Similarly, on a national scale we have estimated societal costs from 3GC-R of close to US$4 million annually by 2030. The impact of the costs of AMR are likely to have the greatest impacts on low-income countries such as Malawi, which have low overall healthcare expenditure, and mitigation strategies all the more important

3. *It is lost the reflexion on the rational use of 3GC in order to improve the health outcomes given the findings (are there some clinical guidelines? is there any surveillance of AMR system?).*

The following text has been added at line 93 of the introduction: The change to these agents circa 2004 was based on ease of administration (once daily) and affordability (donor funded) as well as antimicrobial susceptibilities at the time.
