# Supplementary material for: Individual and population level costs and health-related quality of life outcomes of third-generation cephalosporin resistant bloodstream infection in Blantyre, Malawi
Source: PLOS Glob Public Health. 2023 Jun 22;3(6):e0001589. doi: 10.1371/journal.pgph.0001589 (PMC10287011; doi:10.1371/journal.pgph.0001589)
Supplement: Attachment — Submitted filename: Response to Reviewers_v2.docx [file pgph.0001589.s008.docx]

20^th^ January 2023

Dear Editor

**Manuscript ID: PGPH-D-22-01156R1**

**Manuscript Title: Individual and population level costs and health-related quality of life outcomes of third-generation cephalosporin resistant bloodstream infections in Malawi.**

We have addressed the minor revisions requested by the reviewers and have adjusted the formatting of Table 7

Yours sincerely,

Dr Rebecca Lester (on behalf of the authors)
